# Transcriptome analysis reveals key developmental and metabolic regulatory aspects of oil palm (*Elaeis guineensis* Jacq.) during zygotic embryo development

**DOI:** 10.1186/s12870-022-03459-2

**Published:** 2022-03-12

**Authors:** Anni Zhang, Longfei Jin, Rajesh Yarra, Hongxing Cao, Ping Chen, Jerome Jeyakumar John Martin

**Affiliations:** 1 Key Laboratory for Quality Regulation of Tropical Horticultural Crops of Hainan Province, College of Horticulture, Hainan University, Haikou, 570228 China; 2 Coconut Research Institute, Chinese Academy of Tropical Agricultural Sciences / Hainan Key Laboratory of Tropical Oil Crops Biology, Wenchang, 571339 China

**Keywords:** *Elaeis guineensis*, Embryonic development, RNA-Seq, Phytohormone, Carbohydrate, Sucrose and starch metabolism, Fatty acid biosynthesis

## Abstract

**Background:**

Oil palm is the most efficient oil-producing crop in the world, and the yield of palm oil is associated with embryonic development. However, a comprehensive understanding of zygotic embryo development at the molecular level remains elusive. In order to address this issue, we report the transcriptomic analysis of zygotic embryo development in oil palm, specifically focusing on regulatory genes involved in important biological pathways.

**Results:**

In this study, three cDNA libraries were prepared from embryos at S1 (early-stage), S2 (middle-stage), and S3 (late-stage). There were 16,367, 16,500, and 18,012 genes characterized at the S1, S2, and S3 stages of embryonic development, respectively. A total of 1522, 2698, and 142 genes were differentially expressed in S1 vs S2, S1 vs S3, and S2 vs S3, respectively. Using Gene Ontology (GO) term enrichment and Kyoto Encyclopedia of Genes and Genomes (KEGG) pathway analysis to identify key genes and pathways. In the hormone signaling pathway, genes related to auxin antagonize the output of cytokinin which regulates the development of embryo meristem. The genes related to abscisic acid negatively regulating the synthesis of gibberellin were strongly up-regulated in the mid-late stage of embryonic development. The results were reported the early synthesis and mid-late degradation of sucrose, as well as the activation of the continuous degradation pathway of temporary starch, providing the nutrients needed for differentiation of the embryonic cell. Moreover, the transcripts of genes involved in fatty acid synthesis were also abundantly accumulated in the zygotic embryos.

**Conclusion:**

Taken together, our research provides a new perspective on the developmental and metabolic regulation of zygotic embryo development at the transcriptional level in oil palm.

**Supplementary Information:**

The online version contains supplementary material available at 10.1186/s12870-022-03459-2.

## Background

Oil palm (*Elaeis guineensis* Jacq.) is a tropical woody crop with the highest oil production capability worldwide, accounting for 35% of the global edible vegetable oil production [1]. The increasing demand for biofuels has accelerated the global expansion of oil palm farms [2]. The production of palm oil, on the other hand, is primarily influenced by fruit development, which is dependent on the proper development of zygotic embryos. There is no precise knowledge of the development of zygotic embryos in the oil palm. Somatic embryogenesis is a model for understanding zygotic embryo development. Proembryos, characterized by a collection of thick-walled embryonic cells, are formed at the stage of callus differentiation [3]. In embryogenic cells, starch granules are visible, whereas they are nearly nonexistent in cells of the proembryo, whereby proteins are stored in the form of proembryos, which are then employed for the development of the somatic embryo as a reserve component [4]. Then globular embryos are then produced around meristematic cells. The somatic embryo at the torpedo stage is differentiated into distal regions that include the procambium (vascular tissue), the basic meristem, and the protoderm, and proximal regions consisting of the embryonic axis, which characterize the bipolar axis [5]. Although procambium and fundamental meristem cells lack starch grains, they do have reserve proteins [4]. Finally, the somatic embryos are completely regenerated and continue to develop. These findings are important for the development of oil palm zygotic embryos.

At various developmental stages, hormone regulation, metabolic pathways (sucrose and starch metabolism), as well as fatty acid (FA) biosynthesis play a vital part in morphology and maturation of embryos [6–8]. In particular, a balance of auxin/cytokinin (IAA/CTK) and abscisic acid/gibberellin (ABA/GA) is required to stimulate embryonic maturation [9, 10]. During embryonic development, the preponderance of the carbon flow balance is concentrated in the form of sucrose, starch and FA [11, 12]. Under physiological conditions, the only two essential enzymes for sucrose degradation are sucrose synthase (SUS) and invertase (INV). The SUS gene is activated in the embryo and endosperm when the potato sucrose synthesis gene is over-expressed in cotton, thus enhancing seed set [13]. The functional deficiency mutation in the gene encoding a cell wall invertase (CWIN) causes a substantial reduction in the mass, starch, glucose, and fructose of the embryo [14]. Granule-bound starch synthase (GBSS) and starch synthase (SS) generate amylose and amylopectin, respectively. GBSS has two isoforms: GBSSI and GBSSII. The mutation of GBSSI resulted in waxy maize, which contains the normal amylose content of embryonic starch, as only GBSSII is expressed in the embryo [15]. Furthermore, embryonic starch is transient [16, 17]. The synthesis of starch in the early stages is essential for the accumulation of FA in the later stages of embryonic development. A cDNA encoding the small subunit of glucose-1-phosphate adenylyltransferase (glgC) in the antisense orientation could inhibit starch synthesis and delay lipid formation in the embryo [18]. FA synthesis is important for embryo development. Fatty acyl-ACP thioesterase B (FATB) is involved in free FA release, and the embryo development of *Arabidopsis fatb* knockout mutation is significantly impaired, with distorted seed morphology and a low germination rate [19].

RNA sequencing (RNA-Seq) has the advantages of high sensitivity, digital signal, and a wide detection range. Recently, the gene expression profiles and differentially expressed genes (DEGs) in the embryonic development of various species such as *Arabidopsis*, soybean, maize, and cotton have been widely discovered using RNA-Seq [20–23]. Meanwhile, RNA-Seq has been performed on developing flower and fruit samples of normal and mantled oil palm [24], mesocarp, seed kernel, and selected vegetative tissues [25], and mature oil palm basal trunk tissue infected with pathogen [26] to characterize their transcriptomes. However, research on oil palm zygotic embryos has mostly emphasized on genetic transformation, phytohormones involved in seed germination, and lipid synthesis differences in distinct organs compared to other oil-secretory systems [27–29]. Moreover, transcriptomic changes during embryonic development have not yet been explored. With the availability of oil palm genome sequencing data, it is possible to capture the expression abundance and cleavage of all known genes in a certain biological state through transcriptome analysis. To clarify the molecular mechanisms of embryos from development to maturation and reveal the main regulatory genes involved in the biological pathways, RNA-Seq was used to explore the transcriptomic mechanisms of oil palm zygotic embryos in the early (S1), middle (S2), and late (S3) stages of development. Our findings pave the way for more research into the embryonic development of other oil-producing crops in the future.

## Results

### Transcriptome alignment

To identify transcripts and biological processes during embryonic development, three cDNA libraries were constructed from three stages of zygotic embryo development of oil palm (S1, S2, and S3) and further sequenced. A summary of transcriptome alignment information is given in Table 1. A total of 167,919,201 clean reads were obtained after data cleaning and quality verification. The reads were successfully mapped on the reference genome by 93.91–94.57%, of which 90.09–92.49% were mapped uniquely. In addition, a total of 64,016 known genes and 4246 new genes were found. The randomness assessment showed that the read number mapped to different locations of genes was presented in a normal distribution (Additional File 1: Figure s1). The growth rate of detected genes was flat when it reached 15 × 10^6^ in the saturation graph of transcriptome data, indicating the sequencing of all genes in the sample (Additional File 2: Figure s2).

**Table 1:** The transcriptome sequencing summary in *Elaeis guineensis*

Sample	Raw reads	Clean reads	Unique Mapped (%)	Multiple Mapped (%)	Total Mapped (%)	Known Genes (%)	Novel Genes (%)	Total Genes (%)
S1	67,453,381	66,503,829	61,512,610 (92.49%)	1,395,157 (2.08%)	62,907,767 (94.57%)	20,533 (77.84%)	1417 (95.27%)	21,950 (78.76%)
S2	50,449,958	49,974,160	45,018,868 (90.09%)	1,909,605 (3.82%)	46,928,473 (93.91%)	21,279 (80.66%)	1405 (94.49%)	22,684 (81.40%)
S3	52,439,644	51,441,212	46,794,507 (90.96%)	1,582,612 (3.08%)	48,377,119 (94.04%)	22,204 (84.17%)	1424 (95.79%)	23,629 (84.79%)

### Global analysis of gene expression profiles during embryonic development

**Fig. 1:**
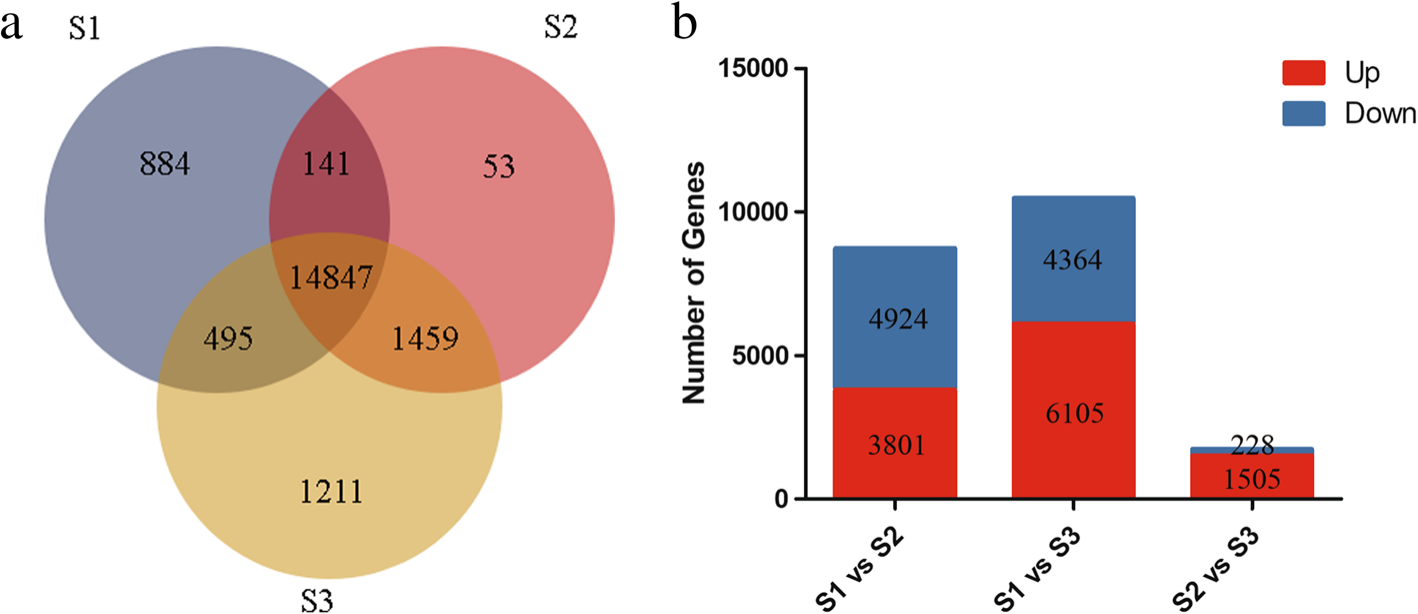
Statistical analysis of DEGs during zygotic embryo development (S1, S2, and S3). **a** Venn diagram of expressed genes detected in three developmental stages. **b** DEGs histogram revealed by pairwise comparisons between S1 vs S2, S1 vs S3, and S2 vs S3

Our study revealed the presence of detectable expression signals (FPKM≥1) in a total of 19,090 genes, as shown in Fig. 1a. Of those, more than 77.77% of the genes were detected at all three developmental stages (S1, S2, and S3). A total of 884 and 1211 genes were found to be expressed in S1 and S3, respectively, while 53 unique genes were found only in the S2 phase, reflecting differences in spatial transcription patterns at different developmental stages. A total of 8225 (S1 vs S2), 10,469 (S1 vs S3), and 1734 (S2 vs S3) DEGs were selected in pairwise comparisons of three developmental stages on the basis of threshold values of FDR < 0.05 and |log2FC| > 1 (Fig. 1b). Compared to stage S1, the stage S2 showed 3801 up-regulated genes and 4924 down-regulated genes, but stage S3 showed only 1505 up-regulated genes and 228 down-regulated genes compared with the stage S2, which indicated the obvious differences between the transcriptome of early and late embryo stages.

### Enrichment analysis of DEGs

More DEGs were involved in “biological process” including ‘metabolic process’ (13.39–16.03%), ‘cellular process’ (12.66–14.82%), and ‘single-organism process’ (9.98–13.84%), mainly distributed in “cellular component” including ‘cell’ (7.20–9.11%), ‘cell part’ (7.20–9.11%), and ‘membrane’ (4.38–5.82%), and “molecular function” including ‘catalytic activity’ (13.35–16.26%), ‘binding’ (9.20–11.19%), and ‘transporter activity’ (1.08–7.73%) according to GO annotation and functional classification (Additional File 3: Figure s3). We showed that the zygotic embryo development of oil palm is comprehensively regulated by the quantities of associated genes, among which DEGs correlated with the “metabolic process” accounted for the most.

**Fig. 2:**
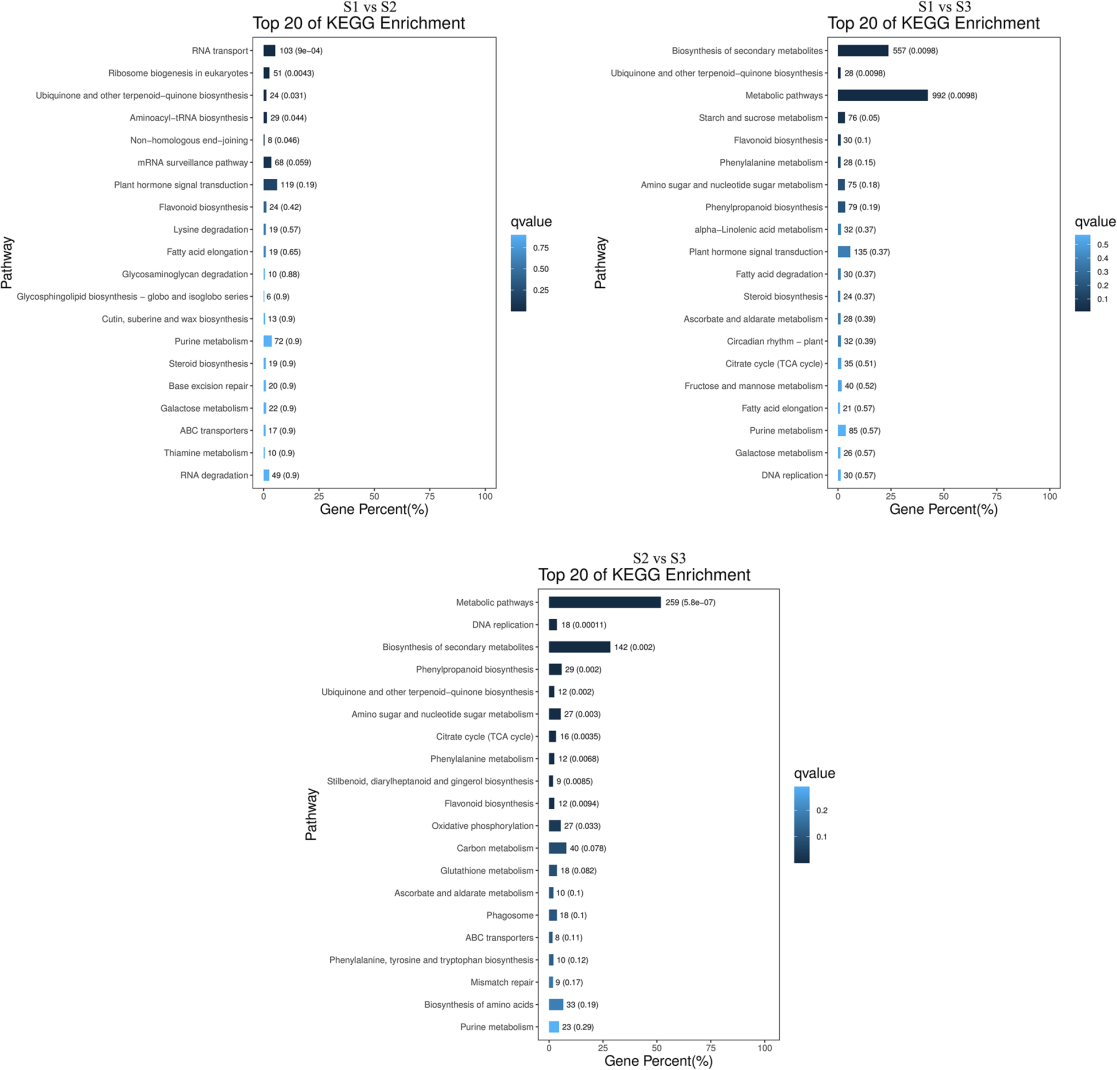
KEGG enrichment classification for the pairwise comparisons of S1 vs S2, S1 vs S3, and S2 vs S3. The color of points represents the q-value, and the darker the color, the smaller the q value. The numbers next to the bar chart are the DEGs and q value mapped to the reference pathway

According to the analysis of the KEGG metabolic pathway, 1968 DEGs (S1 vs S2) were annotated to 132 pathways; 2340 DEGs (S1 vs S3) to 131 pathways, and 500 DEGs (S2 vs S3) to 116 pathways. As shown in Fig. 2, the gene enrichment pathways annotated by the three comparison groups were mainly ‘metabolic pathways’ (38.31, 42.39, and 51.8%, respectively), ‘biosynthesis of secondary metabolites’ (21.85, 23.8, and 28.4%, respectively), ‘plant-pathogen interaction’ (6.15, 7.82, and 8.4%, respectively), and ‘plant hormone signal transduction’ (6.05, 5.77, and 3.8%, respectively). Moreover, dozens of DEGs were related to ‘hormone biosynthesis’, ‘starch and sucrose metabolism’, and ‘FA biosynthesis’. Based on the KEGG analysis, we further studied the specific functional genes related to embryonic development.

### Differential expression analysis of plant hormones signaling pathway-related genes during embryonic development

**Fig. 3:**
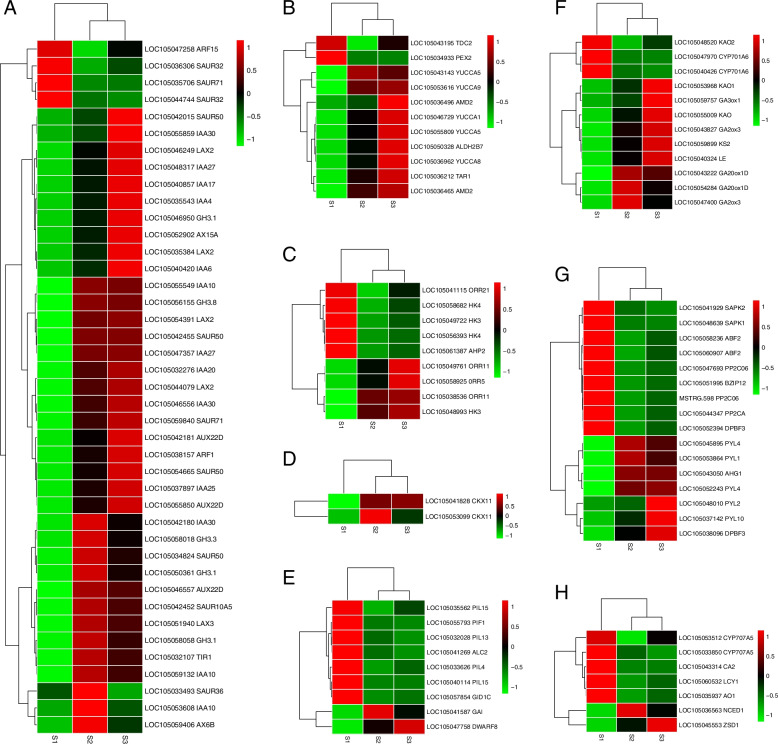
Heatmap of DEGs in the hormone signaling pathway. **a** Auxin signal transduction **b** IAA biosynthesis **c** CTK signal transduction **d** CTK biosynthesis **e** GA signal transduction **f** GA biosynthesis **g** ABA signal transduction **h** ABA biosynthesis. The 3 columns in each heatmap represent the zygotic embryo development stages (S1, S2, and S3). Heatmaps indicate the gene expression level by log2(FPKM) with a rainbow color scale. Each row represents a single gene, the IDs and names of selected DEGs are indicated to the right of the histograms, and each column represents a sample

Various phytohormones are induced during the development of oil palm zygotic embryos. In plant zygotic embryo induction, phytohormones either act alone or in combination with other plant growth regulators. To gain a better understanding of hormone regulation, we focused on DEGs of hormonal signal transduction related to IAA, CTK, GA, and ABA biosynthesis. The gene expression patterns of S2 and S3 were similar according to our results (Fig. 3; Additional file 4: Table S1 a-h). Most genes enriched in the IAA signal transduction pathway were expressed at low rates in S1 and at significant concentrations in S2 and S3 (Fig. 3a). The activity levels of *ARF15* and the three *SAUR* genes decreased considerably from S1 to S2 and exhibited lower expression in S3. On the contrary, the expression levels of six *SAUR*, thirteen *IAA*, five *LAX*, five *GH3*, four *AUX*, one *ARF*, and one *TIR1* were elevated from S1 to S2, and the preponderance of the genes remained high in S3. Notably, *SAUR36*, *IAA10*, and *AX6B* were found to be highly transcribed in S2. Apart from *TDC2*, *PEX2*, and *AMD2*, the remaining genes involved in the IAA synthesis pathway exhibit a substantial increasing trend in expression levels from S1 to S2 and were highly expressed in S3 (Fig. 3b). As shown in (Fig. 3c), *ORR21*, *AHP2*, and three *HK* genes tended to be significantly expressed in S1 but lowly expressed in S2 and S3. Moreover, other genes, such as *ORR11*, *ORR5*, and *HK3* had elevated expression levels from S1 to S2 and were substantially expressed in S3. In S2, two *CKX* genes involved in CTK biosynthesis were strongly expressed, with one of them retaining high transcriptional levels in S3 (Fig. 3d). *PIF1*, *ALC2*, *GID1C*, and four *PIL* genes were strongly activated in the GA signaling pathway in S1, and expression levels of *GAI* and *DWARF8* grew dramatically from S1 to S2 and remained high in S3 (Fig. 3e). In terms of GA biosynthesis, *KAO2* and two *CYP701A6* were significantly observed in S1, whereas *GA3ox1*, *KS2*, *LE*, two *GA20ox1D*, two *KAO*, and two *GA2ox3* transcript levels gradually increased from S1 to S2 and continued in S3 (Fig. 3f). Related to the ABA signaling pathway, *BZIP12*, *DPBF3*, two *ABF2*, two *SAPK*, and three *PP2C* genes were specifically and highly expressed in S1, *AHG1*, and three *PYL* were highly expressed in S2 and S3, and the expression levels of *DPBF* and two *PYL* gradually increased from S1 to S2 and remained high in S3 (Fig. 3g). As shown, *CA2*, *LCY1*, *AO1*, and two *CYP707A5* were transcribed at high levels in S1, and *NCED1* and *ZSD1* were highly expressed in S2 and S3, respectively, which are known to be involved in ABA biosynthesis (Fig. 3h).

### Transcriptional activation of starch and sucrose metabolism genes during embryonic development

**Fig. 4:**
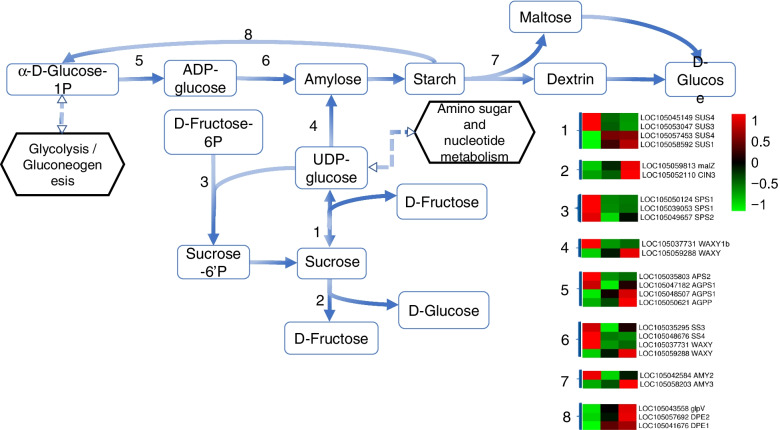
Major metabolic pathways and key enzymes of starch and sucrose metabolism. Transcriptional levels of key genes are represented in heatmaps. Schemes were retrieved from KEGG (ko00500). Corresponding gene numbers are represented as follows with their respective involvement in the pathway indicated: (1) sucrose synthase (SUS); (2) maltase-glucoamylase (MGAM), invertase (INV); (3) sucrose-phosphate synthase (SPS); (4) granule-bound starch synthase (GBSS); (5) glucose-1-phosphate adenylyltransferase (glgC); (6) starch synthase (SS), granule-bound starch synthase (GBSS); (7) alpha-amylase (AMY); (8) starch phosphorylase (PYG). Heatmaps indicate the gene expression level by log2(FPKM) with a rainbow color scale. Each row represents a single gene, the IDs and names of selected DEGs are indicated to the right of the histograms, and each column represents a sample

Our findings revealed 24 DEGs encoding 10 key enzymes, involved in the starch and sucrose metabolic pathways (Fig. 4; Additional file 5: Table S2). During the process of sucrose synthesis, two SUS and three SPS genes were activated in S1, and two SUS genes were primarily mapped in S2 and S3. During the sucrose degradation pathway, the expression levels of one MGAM, one INV, and one GBSS genes were shown to progressively increase from S1 to S3, whereas one GBSS gene was only significantly expressed in S1. Related to the starch synthesis pathway, two glgC, two SS, and one GBSS genes were highly expressed in S1, and two glgC and one GBSS genes were highly expressed in S2 and S3. Furthermore, during the development process, the transcription levels of four PYG and one AMY genes that catalyse starch hydrolysis were gradually increased.

### FA biosynthesis-related genes were highly expressed in the middle and late stages of zygotic embryo development

**Fig. 5:**
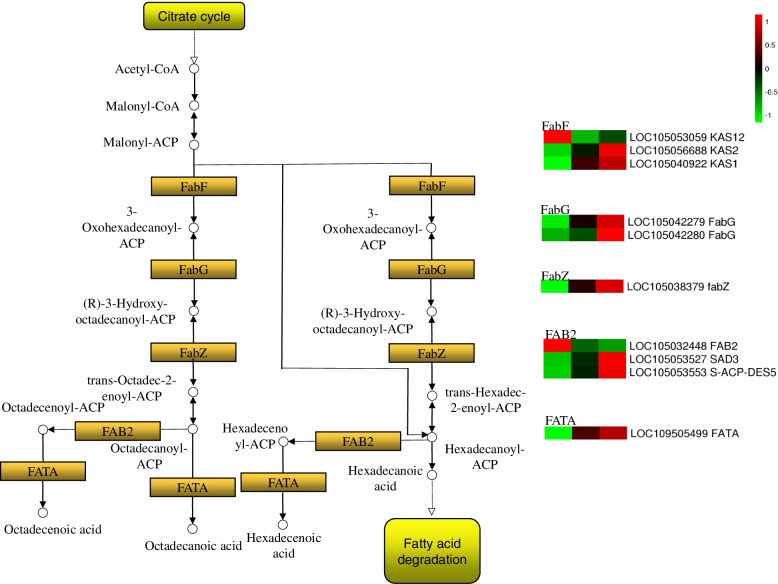
Major fatty acid biosynthesis pathways and key enzymes. Schemes were retrieved from KEGG (ko00061). Shapes and arrows follow the KEGG representation standards (www.kegg.jp/kegg/kegg1.html), except for colour codes. The yellow rectangles represent enzymes. The rounded rectangles represent connected pathways. The white circles represent chemical compound. Heatmaps indicate the gene expression level by log2(FPKM) with a rainbow color scale. Each row represents a single gene, the IDs and names of selected DEGs are indicated to the right of the histograms, and each column represents a sample

A total of 10 DEGs that encode 5 key enzymes were elucidated in the FA synthesis pathway (Fig. 5; Additional file 6: Table S3). Moreover, two genes, i.e., 3-oxoacyl-[acyl-carrier-protein] synthase II (FabF) and acyl-[acyl-carrier-protein] desaturase (FAB2) genes, were mostly transcribed in S1, and their levels declined in S2 and remained low in S3. The expression of two FabF, two 3-oxoacyl-[acyl-carrier protein] reductase (FabG), one 3-hydroxyacyl-[acyl-carrier-protein] dehydratase (FabZ), two FAB2, and one fatty acyl-ACP thioesterase A (FATA) genes increased significantly from S1 to S2 and remained high in S3.

### Relative expression analysis of selected genes during embryonic development

**Fig. 6:**
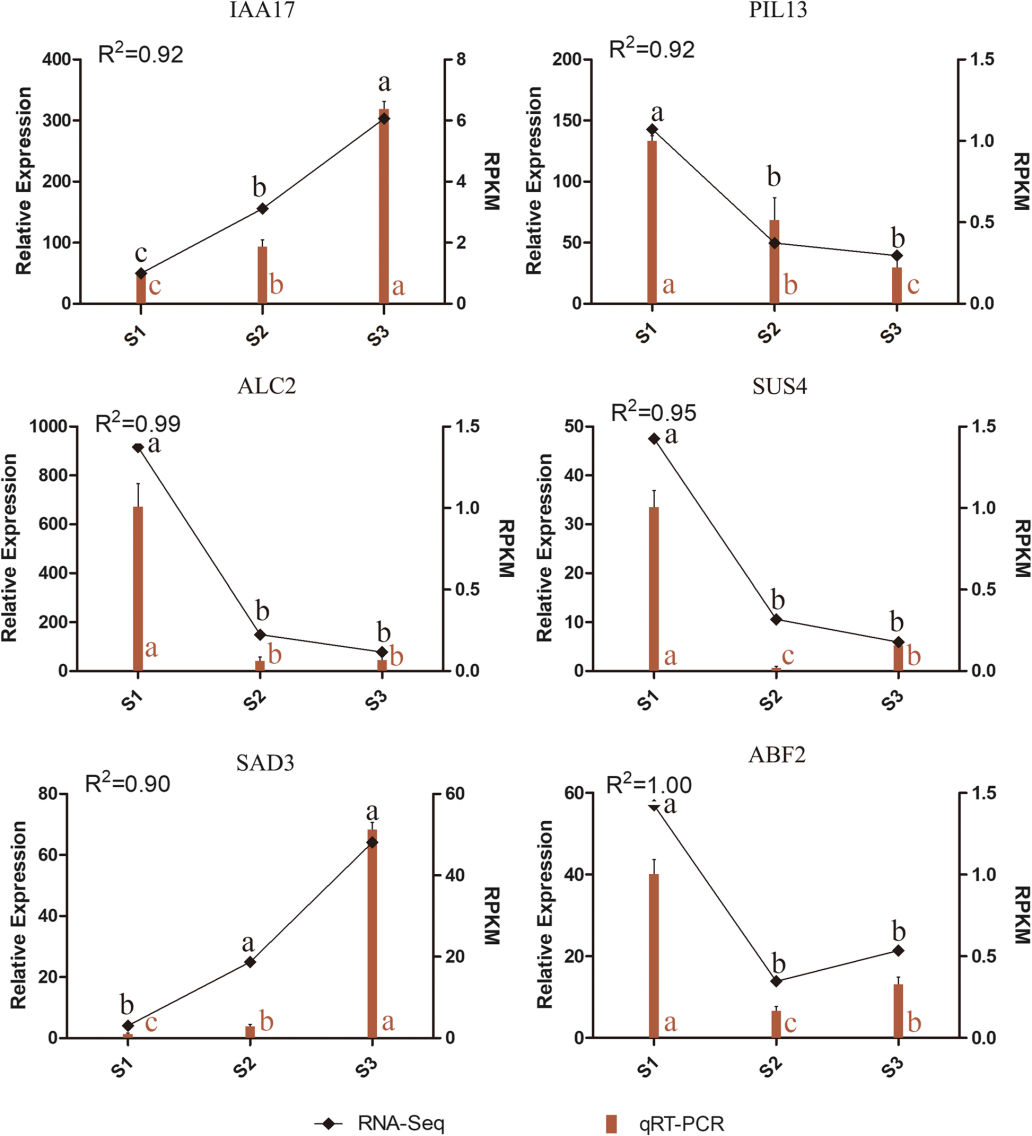
Relative expression levels of six selected genes during zygotic embryonic development (S1, S2, and S3). The 2^-ΔΔCt^ method was used to determine the relative expression levels of genes. The statistical differences were analyzed by ANOVA based on Duncan’s multiple test (*P* < 0.05). Different letters indicate significant differences in the relative expression level and RPKM values

To verify the accuracy of RNA-Seq expression profile sequencing, a total of six DEGs from important metabolic pathways were randomly selected and subjected to qRT-PCR for expression pattern analysis (Fig. 6; Additional file 7: Table S4). The results showed that the qRT-PCR data was trended in line with the RNA-Seq data and the gene decision coefficient (R^2^) exceeded 0.9.

## Discussion

Embryonic development is related to the alternation of generation in plants, and it is vital in defining the formation and diversity of seeds, as well as a range of agronomic traits [30, 31]. With the advent of biotechnology, genes that govern embryonic development have been systematically identified in some model and non-model plants [32, 33]. However, there is no comprehensive overview of the molecular mechanism of zygotic embryo development in oil palm. Significant research in oil palm has been undertaken using transcriptome sequencing technologies to procure an insight into the molecular mechanisms regulating FA biosynthesis [25]. Here, RNA-Seq was used to show transcriptome changes in the early, middle, and late stages of oil palm zygotic embryo development in high throughput and quantitative manner, and the expression patterns of some key genes related to developmental and metabolomic pathways were evaluated.

The signaling pathways of various hormones in plants intersect to form a complex regulatory network, and the dynamic balance of various hormones affects the genesis, and development of embryos. During embryogenesis, the interaction between IAA and CTK regulates meristem development, and interference with the hormone signal transduction pathway will eventually lead to embryonic development damage [34, 35]. The *Arabidopsis* Response Regulator (ARR) is a repressor of CTK signal transduction, and the mutation of *ABPH1* encoding A-type ARR resulted in the decrease of IAA content in the stem meristem of maize embryos and the delay of leaf primordia germination [36]. During *Arabidopsis* root meristem development, IAA induced the formation of an embryonic root stem cell niche by activating *ARR* [10]. Furthermore, at the initial stage of plant root regeneration following a radicle development pattern, the IAA and CTK signal domains briefly overlapped, followed by synergistic up-regulation of CTK (*ARR* family) and IAA (*AUX/IAA* family) response genes [35]. In this study, three *ARR* and thirteen *IAA* genes were significantly expressed in the middle and late stages of embryonic development, which was consistent with a previous finding [20]. Our results substantially confirmed previous findings and revealed that the interaction between IAA and CTK is crucial for the formation and maintenance of the embryo meristem of oil palm.

The antagonism between ABA and GA is important in regulating embryo development, especially because the high ABA/GA ratio contributes to seed germination inhibition and embryo maturation induction during early seed development [37]. Previous reports revealed that the GA biosynthetic genes (*AtGA3ox* and *AtGA20ox*) and GA upregulated genes (*AtEXP2* and *AtCP1*) in the developing seeds of ABA-deficient *Arabidopsis* mutants were highly transcribed, while the inhibition of GA inactivation gene (*AtGA2ox6*) [38]. In this analysis, the expression levels of two *GA2ox*, one *GA3ox*, two *GA20ox*, and two ABA synthesis genes (*NCED1* and *ZSD1*) were high in the S2 and S3 stages of embryonic development, while the expression levels of two ABA degradation genes (*CYP707A5*) decreased significantly in S2 and remained low in S3. But this was different from the previous reports in *Arabidopsis* and barley where expression of GA biosynthetic genes was very low in late embryonic development [38, 39]. However, ABA and GA synthesis occurred simultaneously during cotyledon and hypocotyl development in *Kandelia obovate*, which was a viviparous plant [40]. Oil palm seeds have no dormancy period, and maintaining a high level of GA may promote the embryo to continue to grow and overcome dormancy to directly enter the germination stage. This is consistent with the finding of a continuous strategy of development with a dry condition of the palm *Euterpe edulis* at maturity [41].

Sucrose and starch are the main carbon sources during plant embryo development [12, 42]. Starch and sucrose metabolism in embryos affects seed phenotype [43]. SPS is the main rate-limiting enzyme in sucrose synthesis, which is beneficial for carbohydrate metabolism and distribution during seed development [44]. SPS genes were strongly expressed in the late embryonic development of maize and wheat [45, 46], which was different from the findings of this study, ie., the expression of three SPS genes whose expression was elevated in S1 dropped dramatically in S2 and remained low in S3. This might be related to the maternal tissue supply of photosynthate to seeds [8]. Sucrose could be carried to globular embryos and processed by CWIN in order to give the necessary hexose for cell divisions, hence reducing the requirements of self-synthesized sucrose throughout the middle and late embryonic stages of development [8]. Interestingly, we found that there was a late activation pathway for sucrose degradation, that is, one MGAM, one INV, and two SUS genes were significantly transcribed in the middle and late embryonic stages of development, which was consistent with the previous reports on gene transcription patterns in *Arabidopsis* during embryonic development [47].

Previous studies have shown that the starch accumulated in the embryos of oilseeds was transitory, and the starch content decreased with embryo development [12, 17]. The activity of SS increased in the early embryonic stages of development, while beta-amylase and plastid phosphatase activity increased in the late stages [16]. Similarly, in our study, two SS genes were extremely transcribed in S1 and three PYG genes were strongly transcribed in S2 and S3. However, this study also showed that there was a high expression of amylose synthase (GBSS) and starch hydrolase (AMY) genes throughout the development of oil palm embryos, which was also different from the previous reports [48, 49]. Changes in starch content in embryos are balanced between synthesis and degradation during the development process [16]. As a result, the exploration implies that GBSS and AMY were responsible for the synthesis and degradation of transitory starch in the embryonic development phase, while SS and PYG were responsible for starch synthesis in the early embryo and starch degradation in the late embryo, respectively, to provide nutrients for cell differentiation.

FA is an important storage substance in embryos. It has been proved by reverse genetics that knockout of genes related to FA accumulation can lead to embryo mortality or a potentially significant maternal impact on seed development [19, 50, 51]. In Jatropha, the expression levels of main FA synthase genes such as *FabF* and *FATA* genes peaked in the middle and late embryonic stages of development in high oil content genotypes and exhibited a low expression trend in low oil content genotypes [52]. On the contrary, these genes were discovered to be highly transcribed in the early developing pecan embryo [53]. In this study, they had a coordinated temporal expression pattern, because they were strongly activated in the mid-late periods of embryonic development. Variety differences or differences in major oil-producing organs of species may be the key to the transcriptional regulation differences of FA synthesis in embryos. The results suggested that the FA synthesis pathway was triggered in S3 phase embryonic development of the oil palm, which might be related to a transition in carbon distribution, i.e., increased pyruvate transporter activity promoted an increase in carbon flux required for FA synthesis while decreasing carbon supply for starch synthesis [11, 12].

## Conclusion

In conclusion, our study provides changes in transcription accumulation during the early development to maturity of oil palm zygotic embryos. The KEGG enrichment analysis of DEGs at different stages revealed the dynamic metabolic processes essential for embryo development, including the interaction of plant hormones, starch, and sucrose metabolism, and FA biosynthesis. Several IAA and CTK rapid response genes were strongly transcribed throughout the mid-late stages of embryonic development, implying that IAA negatively regulated CTK signal transduction to maintain meristem development. Furthermore, genes related to ABA synthesis, GA synthesis, and decomposition were active, indicating that ABA antagonised GA to stimulate embryonic maturation and a high GA/ABA ratio was established during embryonic maturation to overcome dormancy. In addition, sucrose metabolism-related genes follow the transcription rules of early synthesis and late degradation of sucrose, and starch reserves show continuous degradation. Finally, we also found a unique expression pattern of genes that regulate FA storage that were transcribed in the mid-late embryonic development. Here, we provide new insight into the embryonic mechanisms of oil palm and lay the foundation for further exploration of the decisive genes of embryonic development.

## Methods

### Plant material

All methods were performed in accordance with the relevant guidelines and regulation. The zygotic embryos were collected from seeds of oil palm (*Elaeis Guineenis* Jacq. cultivar Reyou No.4) after flowering for 120(S1), 140(S2), and 160(S3) days at the National Tropical Palm Germplasm Resource Nursery, Wenchang, Hainan Province, China (110.8° latitude, 19.6° longitude), and then quickly placed in liquid nitrogen for storage. The total RNA was extracted from the S1, S2, and S3 stage zygotic embryos using Trizol reagent (Invitrogen, CA, USA), with 3 biological replicates per developmental stage and each replicate included 27 to 50 embryos. The RNA quality was evaluated using the NanoDrop 2000 spectrophotometer (Thermo Fisher Scientific, Waltham, USA) and RNA integrity was analyzed by Agilent 2100 bioanalyzer (Agilent, CA, USA).

### Library preparation, Illumina sequencing, and data processing

After being enriched by magnetic Oligo (dT) beads, the mRNA was fragmented and a cDNA library was prepared. The RNA sequencing was done at Gene Denovo Biotechnology Company (Guangzhou, China) via Illumina HiSeqTM 2500 platform. The raw data were processed with fastp to obtain clean reads [54]. The Bowtie2 [55] and HISAT2 [56] tools were performed to conduct sequence alignment analysis based on the ribosome database and genome database in turn. The reference genome of oil palm was downloaded from NCBI (https://www.ncbi.nlm.nih.gov/assembly/GCF_000442705.1).

### Screening and functional annotation of DEGs

The gene expression level of three samples was expressed as original reads count and fragments per kilobase of transcript per million mapped reads (FPKM) [57]. Gene expression with FPKM≥1 was considered “expressed”. The DESeq2 software was used to perform pairwise comparisons between three stages (S1 vs S2, S2 vs S3, and S1 vs S3), and DEGs were identified with the false discovery rate (FDR) < 0.05 and the |log2 fold change| > 1 (|log2FC| > 1) [58].

The DEGs were mapped to each term in the Gene Ontology (GO) database (http://www.geneontology.org/) and the number of genes for each term was calculated. The hypergeometric test was used to identify GO items significantly enriched in DEGs compared with the whole genome background. The Kyoto Encyclopedia of Genes and Genomes (KEGG) [59] (http://www.kegg.jp/kegg/pathway.html) enrichment analysis was used to determine the pathway of significant enrichment in DEGs compared with genomic background by applying the hypergeometric test.

### Quantitative real-time PCR

The first-strand cDNA was synthesized from 1.25 μg of total RNA extracted from zygotic embryos at three developmental stages to verify RNA-Seq data by using *EasyScript*® First-Strand cDNA Synthesis SuperMix kit (TransGen, Beijing, China). Six genes with potential roles during embryonic development were further selected for quantitative RT-PCR (qRT-PCR) analysis. The qRT-PCR primers (Table 2) were designed by using the Primer-BLAST online tool (https://www.ncbi.nlm.nih.gov/tools/primer-blast/index.cgi). The relative expression of selected genes was measured in 384-well microplates using ABI QuantStudio™6 Flex quantitative real-time PCR instrument (Thermo Fisher, Waltham, USA) using SYBR® Select Master Mix (Thermo Fisher Scientific, Waltham, USA). The qRT-PCR amplification reactions were performed as follows: 50 °C for 2 min, next DNA polymerase and UP were activated at 95 °C for 2 min, then 95 °C annealing for 15 s, 60 °C extensions for 1 min, and 40 cycles of amplification. Three biological replicates with three technical repeats were performed for the reliability of this experiment. The relative expression of selected genes was calculated using the 2^-ΔΔCt^ method. *Actin* being used as a reference gene [60]. The Duncan’s multiple range test of SAS 9.1 (SAS Institute, Cary, NC, USA) software was used for the significance test (*p* < 0.05).

**Table 2:** Candidate genes selected for oil palm tissue evaluation

Gene symbol	Gene name	Sequence (5′-3′)		Product length
		Forward primer	Reverse primer	
LOC105040857	*IAA17*	ATGCAATTGGACTTGCTCCAAGG	ATGGTCCCTTACAGCCATCTG	110
LOC105032028	*PIL13*	AGCAGAATCCGACAACAGCA	GTGAACCATTTGCAAGATCCAGA	109
LOC105041269	*ALC2*	TTCAGCGATCACCCAGCAAG	AGCCTCCATTTGCATGACGA	83
LOC105058236	*ABF2*	GTGCACGTAAGCAGGCCTAT	TGCATCTCCATCATCTCCTCC	106
LOC105045149	*SUS4*	TCAACGTCTTGAAAGAATTTCTGG	ATTTCACTAGCCGCATCCTCG	150
LOC105053527	*SAD3*	TCCCACCCCATAACCTTTCTTG	GTCATTCTTTACCTCCCTAGGCT	122

## Supplementary Information

**Additional file 1: Figure S1.** Randomness assessments of the three libraries.

**Additional file 2: Figure S2.** Sequencing saturation analysis of the three libraries.

**Additional file 3: Figure S3.** GO enrichment classification histogram for the pairwise comparisons of S1 vs S2, S1 vs S3, and S2 vs S3.

**Additional file 4: Table S1.** Differentially expressed genes involved in hormone signaling pathway during zygotic embryo development of oil palm. S1-a. Differentially expressed genes involved in auxin signalling pathway during zygotic embryo development of oil palm. S1-b. Differentially expressed genes involved in IAA biosynthesis during zygotic embryo development of oil palm. S1-c. Differentially expressed genes involved in CTK signal transduction during zygotic embryo development of oil palm. S1-d. Differentially expressed genes involved in CTK biosynthesis during zygotic embryo development of oil palm. S1-e. Differentially expressed genes involved in GA signal transduction during zygotic embryo development of oil palm. S1-f. Differentially expressed genes involved in GA biosynthesis during zygotic embryo development of oil palm. S1-g. Differentially expressed genes involved in ABA signal transduction during zygotic embryo development of oil palm. S1-h. Differentially expressed genes involved in ABA signal transduction during zygotic embryo development of oil palm.

**Additional file 5: Table S2.** Differentially expressed genes involved in starch and sucrose metabolism during zygotic embryo development of oil palm.

**Additional file 6: Table S3.** Differentially expressed genes involved in fatty acid biosynthesis during zygotic embryo development of oil palm.

**Additional file 7: Table S4.** Comparing differential expression genes from RNA-seq and qRT-PCR during zygotic embryo development of oil palm.

## Data Availability

The data of this project were available at NCBI Sequence Read Archive (SRA): SRP265717 (https://www.ncbi.nlm.nih.gov/sra/?term=SRP265717). All the supporting data are included in Additional files.

## Abbreviations

ABA: Abscisic acid
AMY: Alpha-amylase
ARR: Arabidopsis Response Regulator
CTK: Cytokinin
CWIN: Cell wall invertase
DEGs: Differentially expressed genes
FA: Fatty acid
FAB2: Acyl- [acyl-carrier-protein] desaturase
FabF: 3-oxoacyl-[acyl-carrier-protein] synthase II
FabG: 3-oxoacyl-[acyl-carrier protein] reductase
FabZ: 3-hydroxyacyl-[acyl-carrier-protein] dehydratase
FATA: Fatty acyl-ACP thioesterase A
FATB: Fatty acyl-ACP thioesterase B
FC: Fold change
FDR: False discovery rate
FPKM: Fragments per kilobase of transcript per million mapped reads
GA: Gibberellin
GBSS: Granule-bound starch synthase
glgC: Glucose-1-phosphate adenylyltransferase
GO: Gene Ontology
IAA: Auxin
INV: Invertase
KEGG: Kyoto Encyclopedia of Genes and Genomes
MGAM: Maltase-glucoamylase
PYG: Starch phosphorylase
qRT-PCR: Quantitative real-time PCR
R2: Decision coefficient
RNA-Seq: RNA sequencing
SPS: Sucrose-phosphate synthase
SS: Starch synthase
SUS: Sucrose synthase
