# Supplementary figures and images for: Transcriptome analysis reveals key developmental and metabolic regulatory aspects of oil palm (Elaeis guineensis Jacq.) during zygotic embryo development
Source: BMC Plant Biol. 2022 Mar 12;22:112. doi: 10.1186/s12870-022-03459-2 (PMC8917659; doi:10.1186/s12870-022-03459-2)

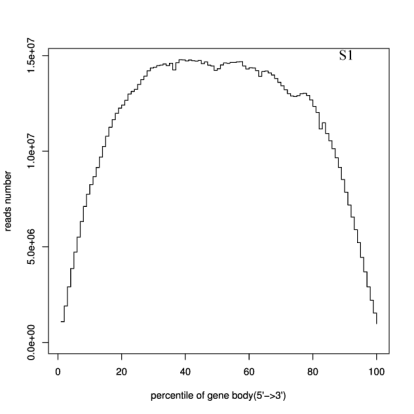

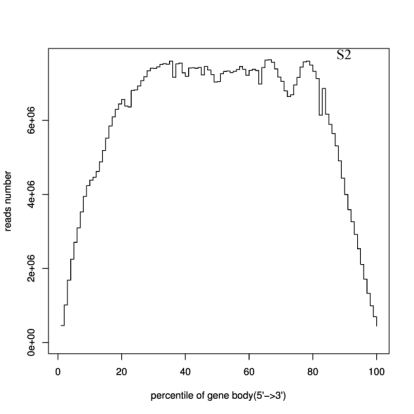


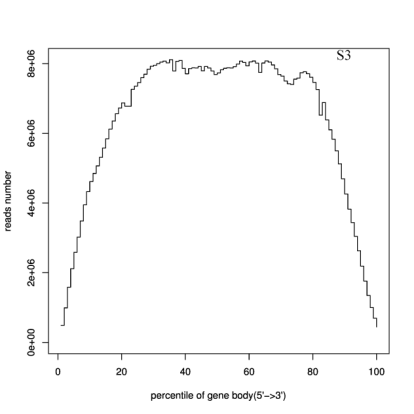

Supplement: Supplementary file 1 — Additional file 1: Figure S1. Randomness assessments of the three libraries. [file 12870_2022_3459_MOESM1_ESM.docx]

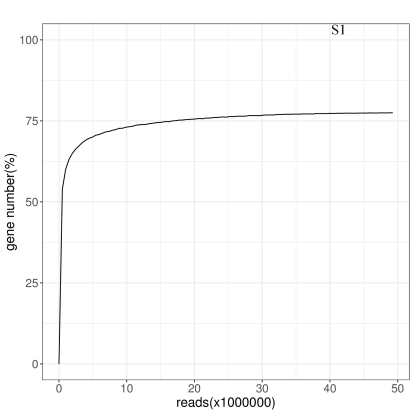

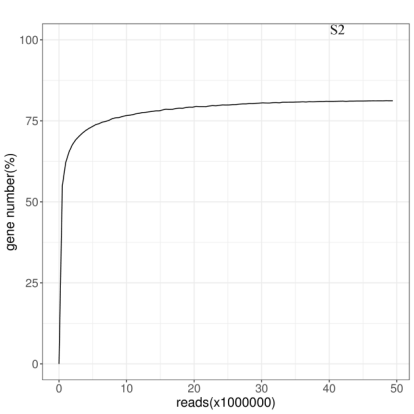


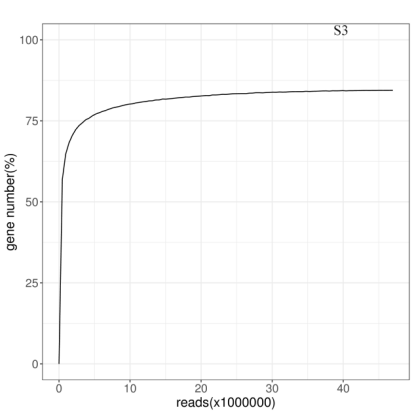

Supplement: Supplementary file 2 — Additional file 2: Figure S2. Sequencing saturation analysis of the three libraries. [file 12870_2022_3459_MOESM2_ESM.docx]

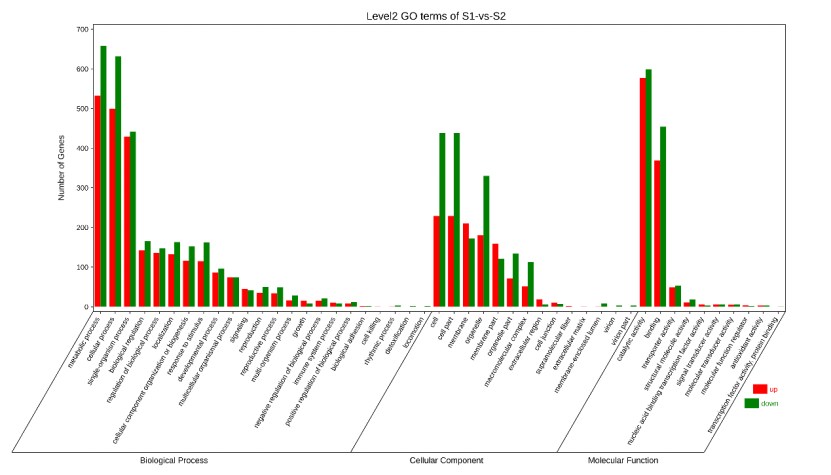

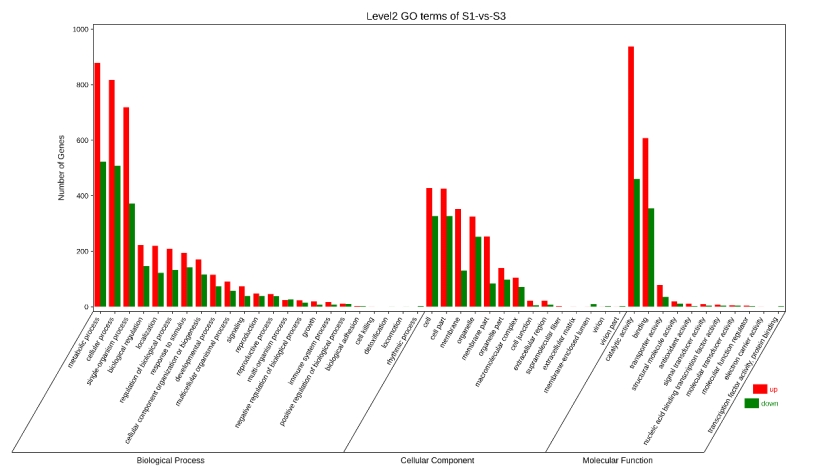

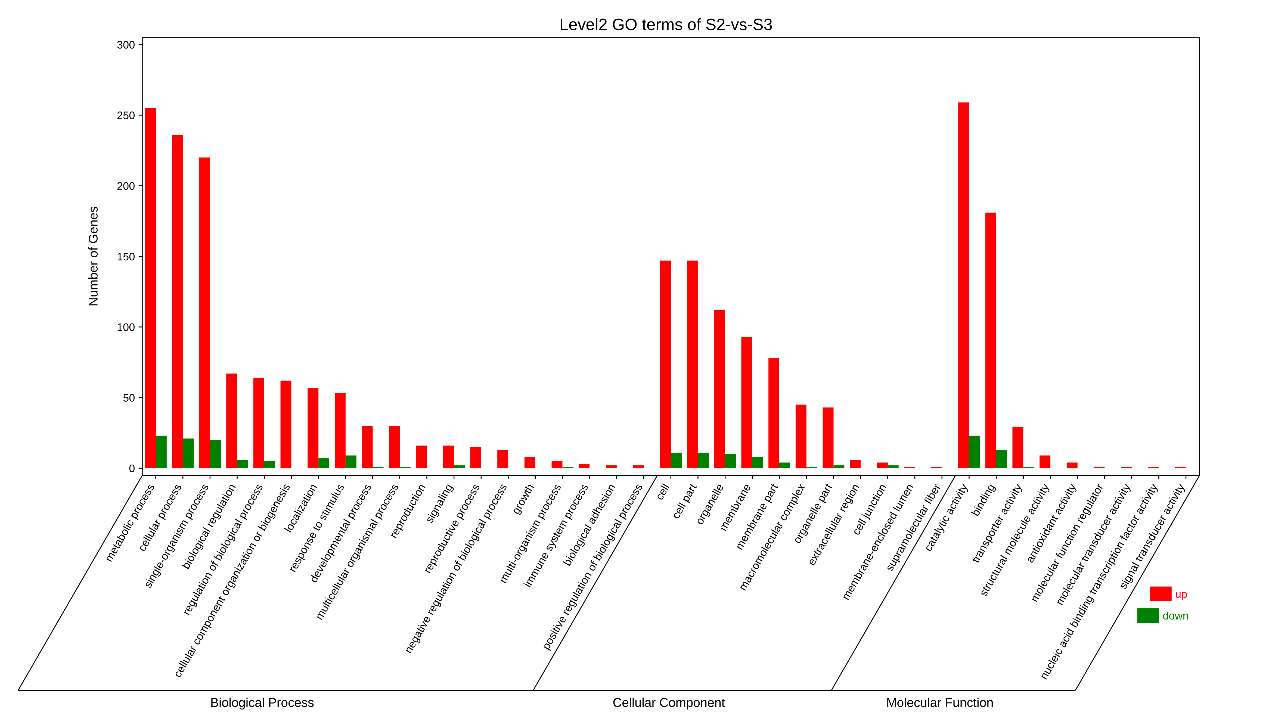

Supplement: Supplementary file 3 — Additional file 3: Figure S3. GO enrichment classification histogram for the pairwise comparisons of S1 vs S2, S1 vs S3, and S2 vs S3. [file 12870_2022_3459_MOESM3_ESM.docx]
