# Supplementary material for: Transcriptome analysis reveals key developmental and metabolic regulatory aspects of oil palm (Elaeis guineensis Jacq.) during zygotic embryo development
Source: BMC Plant Biol. 2022 Mar 12;22:112. doi: 10.1186/s12870-022-03459-2 (PMC8917659; doi:10.1186/s12870-022-03459-2)
Supplement: Supplementary file 4 — Additional file 4: Table S1. Differentially expressed genes involved in hormone signaling pathway during zygotic embryo development of oil palm. S1-a. Differentially expressed genes involved in auxin signalling pathway during zygotic embryo development of oil palm. S1-b. Differentially expressed genes involved in IAA biosynthesis during zygotic embryo development of oil palm. S1-c. Differentially expressed genes involved in CTK signal transduction during zygotic embryo development of oil palm. S1-d. Differentially expressed genes involved in CTK biosynthesis during zygotic embryo development of oil palm. S1-e. Differentially expressed genes involved in GA signal transduction during zygotic embryo development of oil palm. S1-f. Differentially expressed genes involved in GA biosynthesis during zygotic embryo development of oil palm. S1-g. Differentially expressed genes involved in ABA signal transduction during zygotic embryo development of oil palm. S1-h. Differentially expressed genes involved in ABA signal transduction during zygotic embryo development of oil palm. [file 12870_2022_3459_MOESM4_ESM.docx]

| **Additional file 4: Table S1-a** Differentially expressed genes involved in auxin signalling pathway during zygotic embryo development of oil palm. | | | | | | | |
| --- | --- | --- | --- | --- | --- | --- | --- |
| Gene ID | Gene name | S1_FPKM | S2_FPKM | S3_FPKM | S1_*vs*_S2 | S2_*vs*_S3 | Description |
|  |  |  |  |  | Log2 (Fold_change) | Log2 (Fold_change) |  |
| LOC105047258 | *ARF15* | 37.85333333 | 13.52333333 | 25.17 | -1.48 | – | auxin response factor |
| LOC105036306 | *SAUR32* | 17.32 | 4.993333333 | 7.816666667 | -1.79 | – | auxin-responsive protein SAUR |
| LOC105035706 | *SAUR71* | 5.286666667 | 1.91 | 2.023333333 | -1.47 | – | auxin-responsive protein SAUR |
| LOC105044744 | *SAUR32* | 1.8 | 0.103333333 | 0 | -4.12 | – | auxin-responsive protein SAUR |
| LOC105042015 | *SAUR50* | 0.036666667 | 0.576666667 | 2.15 | 3.98 | – | auxin-responsive protein SAUR |
| LOC105055859 | *IAA30* | 1.816666667 | 6.92 | 23.12333333 | 1.93 | 1.74 | auxin-responsive protein IAA |
| LOC105046249 | *LAX2* | 0.873333333 | 3.773333333 | 6.946666667 | 2.11 | – | auxin transporter-like protein |
| LOC105048317 | *IAA27* | 7.066666667 | 69.47333333 | 147.9233333 | 3.30 | – | auxin-responsive protein IAA |
| LOC105040857 | *IAA17* | 49.66666667 | 155.7366667 | 303.17 | 1.65 | – | auxin-responsive protein IAA |
| LOC105035543 | *IAA4* | 0 | 0.2 | 0.493333333 | 7.64 | – | auxin-responsive protein IAA |
| LOC105046950 | *GH3.1* | 0 | 0.626666667 | 1.556666667 | 9.29 | – | indole-3-acetic acid-amido synthetase |
| LOC105052902 | *AX15A* | 0 | 0.536666667 | 1.333333333 | 9.07 | – | auxin-induced protein |
| LOC105035384 | *LAX2* | 0.033333333 | 0.2 | 0.48 | 2.58 | – | auxin transporter-like protein |
| LOC105040420 | *IAA6* | 0.203333333 | 0.683333333 | 1.6 | 1.75 | – | auxin-responsive protein IAA |
| LOC105055549 | *IAA10* | 1.44 | 16.8 | 16.25 | 3.54 | – | auxin-responsive protein IAA |
| LOC105056155 | *GH3.8* | 0.09 | 45.56666667 | 43.37333333 | 8.98 | – | indole-3-acetic acid-amido synthetase |
| LOC105054391 | *LAX2* | 0.026666667 | 15.32666667 | 16.32333333 | 9.17 | – | auxin transporter-like protein |
| LOC105042455 | *SAUR50* | 0.013333333 | 2.103333333 | 2.113333333 | 7.30 | – | auxin-responsive protein SAUR |
| LOC105047357 | *IAA27* | 2.573333333 | 10.78666667 | 11.05 | 2.07 | – | auxin-responsive protein IAA |
| LOC105032276 | *IAA20* | 7.666666667 | 78.38666667 | 99.18333333 | 3.35 | – | auxin-responsive protein IAA |
| LOC105044079 | *LAX2* | 2.23 | 16.41333333 | 20.58666667 | 2.88 | – | auxin transporter-like protein |
| LOC105046556 | *IAA30* | 0.153333333 | 1.46 | 1.943333333 | 3.25 | – | auxin-responsive protein IAA |
| LOC105059840 | *SAUR71* | 6.08 | 12.88 | 15.78 | 1.08 | – | auxin-responsive protein SAUR |
| LOC105042181 | *AUX22D* | 0.006666667 | 0.63 | 1.19 | 6.56 | – | auxin-induced protein |
| LOC105038157 | *ARF1* | 2.076666667 | 5.363333333 | 7.826666667 | 1.37 | – | auxin response factor |
| LOC105054665 | *SAUR50* | 0.11 | 1.666666667 | 2.846666667 | 3.92 | – | auxin-responsive protein SAUR |
| LOC105037897 | *IAA25* | 0.016666667 | 0.526666667 | 0.883333333 | 4.98 | – | auxin-responsive protein IAA |
| LOC105055850 | *AUX22D* | 0.57 | 1.523333333 | 2.156666667 | 1.42 | – | auxin-induced protein |
| LOC105042180 | *IAA30* | 0.05 | 0.373333333 | 0.223333333 | 2.90 | – | auxin-responsive protein IAA |
| LOC105058018 | *GH3.3* | 0 | 0.5 | 0.273333333 | 8.97 | – | indole-3-acetic acid-amido synthetase |
| LOC105034824 | *SAUR50* | 4.443333333 | 9.65 | 7.676666667 | 1.12 | – | auxin-responsive protein SAUR |
| LOC105050361 | *GH3.1* | 0 | 0.126666667 | 0.073333333 | 6.98 | – | indole-3-acetic acid-amido synthetase |
| LOC105046557 | *AUX22D* | 0.036666667 | 2.706666667 | 2.176666667 | 6.21 | – | auxin-induced protein |
| LOC105042452 | *SAUR10A5* | 2.813333333 | 6.853333333 | 5.9 | 1.28 | – | auxin-responsive protein SAUR |
| LOC105051940 | *LAX3* | 0.006666667 | 0.99 | 0.756666667 | 7.21 | – | auxin transporter-like protein |
| LOC105058058 | *GH3.1* | 0.163333333 | 3.94 | 2.72 | 4.59 | – | indole-3-acetic acid-amido synthetase |
| LOC105032107 | *TIR1* | 65.71666667 | 139.0366667 | 119.3566667 | 1.08 | – | transport inhibitor response 1-like protein |
| LOC105059132 | *IAA10* | 7.043333333 | 24.31 | 19.41333333 | 1.79 | – | auxin-responsive protein IAA |
| LOC105033493 | *SAUR36* | 0.19 | 0.5 | 0.113333333 | 1.40 | -2.14 | auxin-responsive protein SAUR |
| LOC105053608 | *IAA10* | 45.28 | 99.96 | 56.21 | 1.14 | – | auxin-responsive protein IAA |
| LOC105059406 | *AX6B* | 1.623333333 | 5.98 | 2.996666667 | 1.88 | – | auxin-induced protein |

| **Additional file 4: Table S1-b** Differentially expressed genes involved in IAA biosynthesis during zygotic embryo development of oil palm. | | | | | | | |
| --- | --- | --- | --- | --- | --- | --- | --- |
| Gene ID | Gene name | S1_FPKM | S2_FPKM | S3_FPKM | S1_*vs*_S2 | S2_*vs*_S3 | Description |
|  |  |  |  |  | Log2 (Fold_change) | Log2 (Fold_change) |  |
| LOC105043195 | *TDC2* | 0.99 | 0.31 | 0.72 | -1.66 | – | tryptophan decarboxylase |
| LOC105034933 | *PEX2* | 23.14 | 9.46 | 9.31 | -1.29 | – | pollen-specific leucine-rich repeat extensin-like protein |
| LOC105043143 | *YUCCA5* | 0.00 | 0.18 | 0.15 | 7.49 | – | indole-3-pyruvate monooxygenase |
| LOC105053616 | *YUCCA9* | 0.00 | 0.20 | 0.23 | 7.67 | – | indole-4-pyruvate monooxygenase |
| LOC105036496 | *AMD2* | 0.01 | 0.40 | 1.86 | 5.32 | – | amidase |
| LOC105046729 | *YUCCA1* | 6.81 | 16.80 | 25.75 | 1.30 | – | indole-3-pyruvate monooxygenase |
| LOC105055809 | *YUCCA5* | 0.00 | 0.11 | 0.21 | 6.74 | – | indole-4-pyruvate monooxygenase |
| LOC105050328 | *ALDH2B7* | 4.04 | 24.02 | 45.44 | 2.57041888 | – | aldehyde dehydrogenase family |
| LOC105036962 | *YUCCA8* | 0.00 | 0.41 | 0.88 | 8.67 | – | indole-3-pyruvate monooxygenase |
| LOC105036212 | *TAR1* | 2.16 | 7.15 | 10.63 | 1.73 | – | tryptophan aminotransferase-related protein |
| LOC105036465 | *AMD2* | 0.00 | 0.13 | 0.18 | 6.98 | – | amidase |

| **Additional file 4: Table S1-c** Differentially expressed genes involved in CTK signal transduction during zygotic embryo development of oil palm. | | | | | | | |
| --- | --- | --- | --- | --- | --- | --- | --- |
| Gene ID | Gene name | S1_FPKM | S2_FPKM | S3_FPKM | S1_*vs*_S2 | S2_*vs*_S3 | Description |
|  |  |  |  |  | Log2 (Fold_change) | Log2 (Fold_change) |  |
| LOC105041115 | *ORR21* | 0.31 | 0.03 | 0.14 | -3.37 | – | two-component response regulator |
| LOC105058682 | *HK4* | 35.37 | 10.62 | 16.80 | -1.74 | – | histidine kinase |
| LOC105049722 | *HK3* | 13.21 | 4.18 | 5.72 | -1.66 | – | histidine kinase |
| LOC105056393 | *HK4* | 10.13 | 3.25 | 4.06 | -1.64 | – | histidine kinase |
| LOC105061387 | *AHP2* | 39.74 | 10.25 | 14.06 | -1.96 | – | histidine-containing phosphotransfer protein |
| LOC105049761 | *ORR11* | 0.05 | 0.47 | 0.95 | 3.13 | – | two-component response regulator |
| LOC105058925 | *0RR5* | 0.41 | 6.04 | 12.92 | 3.89 | – | two-component response regulator |
| LOC105038536 | *ORR11* | 0.07 | 0.46 | 0.51 | 2.78 | – | two-component response regulator |
| LOC105048993 | *HK3* | 0.10 | 0.44 | 0.53 | 2.20 | – | histidine kinase |

| **Additional file 4: Table S1-d** Differentially expressed genes involved in CTK biosynthesis during zygotic embryo development of oil palm. | | | | | | | |
| --- | --- | --- | --- | --- | --- | --- | --- |
| Gene ID | Gene name | S1_FPKM | S2_FPKM | S3_FPKM | S1_*vs*_S2 | S2_*vs*_S3 | Description |
|  |  |  |  |  | Log2 (Fold_change) | Log2 (Fold_change) |  |
| LOC105041828 | *CKX11* | 0.42 | 3.16 | 3.29 | 2.92 | – | cytokinin dehydrogenase |
| LOC105053099 | *CKX11* | 0.00 | 0.10 | 0.03 | 6.69 | – | cytokinin dehydrogenase |

| **Additional file 4: Table S1-e** Differentially expressed genes involved in GA signal transduction during zygotic embryo development of oil palm. | | | | | | | |
| --- | --- | --- | --- | --- | --- | --- | --- |
| Gene ID | Gene name | S1_FPKM | S2_FPKM | S3_FPKM | S1_*vs*_S2 | S2_*vs*_S3 | Description |
|  |  |  |  |  | Log2 (Fold_change) | Log2 (Fold_change) |  |
| LOC105035562 | *PIL15* | 4.93 | 1.51 | 2.39 | -1.71 | – | transcription factor phytochrome interacting factor-like |
| LOC105055793 | *PIF1* | 77.16 | 10.30 | 6.73 | -2.90 | – | phytochrome-interacting factor |
| LOC105032028 | *PIL13* | 142.86 | 49.73 | 39.38 | -1.52 | – | transcription factor phytochrome interacting factor-like |
| LOC105041269 | *ALC2* | 916.42 | 149.37 | 77.78 | -2.62 | – | phytochrome-interacting factor |
| LOC105033626 | *PIL4* | 1.92 | 0.75 | 0.90 | -1.35 | – | phytochrome-interacting factor |
| LOC105040114 | *PIL15* | 9.89 | 2.35 | 2.46 | -2.07 | – | transcription factor phytochrome interacting factor-like |
| LOC105057854 | *GID1C* | 11.45 | 4.69 | 5.09 | -1.29 | – | gibberellin receptor |
| LOC105041587 | *GAI* | 8.73 | 20.44 | 14.12 | 1.23 | – | DELLA protein |
| LOC105047758 | *DWARF8* | 0.97 | 2.80 | 4.18 | 1.52 | – | DELLA protein |

| **Additional file 4: Table S1-f** Differentially expressed genes involved in GA biosynthesis during zygotic embryo development of oil palm. | | | | | | | |
| --- | --- | --- | --- | --- | --- | --- | --- |
| Gene ID | Gene name | S1_FPKM | S2_FPKM | S3_FPKM | S1_*vs*_S2 | S2_*vs*_S3 | Description |
|  |  |  |  |  | Log2 (Fold_change) | Log2 (Fold_change) |  |
| LOC105048520 | *KAO2* | 14.42 | 6.41 | 8.80 | -1.17 | – | ent-kaurenoic acid oxidase |
| LOC105047970 | *CYP701A6* | 15.62 | 1.29 | 1.26 | -3.60 | – | ent-kaurene oxidase |
| LOC105040426 | *CYP701A6* | 74.32 | 21.45 | 17.38 | -1.79 | – | ent-kaurene oxidase |
| LOC105053968 | *KAO1* | 0.01 | 0.39 | 1.17 | 5.30 | – | ent-kaurenoic acid oxidase |
| LOC105059757 | *GA3ox1* | 0.01 | 0.26 | 1.02 | 4.30 | – | gibberellin 3-beta-dioxygenase |
| LOC105055009 | *KAO* | 0.01 | 0.24 | 0.53 | 4.56 | – | ent-kaurenoic acid oxidase |
| LOC105043827 | *GA2ox3* | 0.45 | 1.91 | 3.01 | 2.07 | – | gibberellin 2-beta-dioxygenase |
| LOC105059899 | *KS2* | 1.69 | 3.48 | 5.14 | 1.04 | – | ent-kaur-16-ene synthase |
| LOC105040324 | *LE* | 0.00 | 0.41 | 0.77 | 8.67 | – | gibberellin 3-beta-dioxygenase |
| LOC105043222 | *GA20ox1D* | 0.38 | 5.96 | 4.91 | 3.98 | – | gibberellin 20 oxidase |
| LOC105054284 | *GA20ox1D* | 0.01 | 0.45 | 0.25 | 6.07 | – | gibberellin 20 oxidase |
| LOC105047400 | *GA2ox3* | 0.00 | 0.32 | 0.16 | 8.34 | – | gibberellin 2-beta-dioxygenase |

| **Additional file 4: Table S1-g** Differentially expressed genes involved in ABA signal transduction during zygotic embryo development of oil palm. | | | | | | | |
| --- | --- | --- | --- | --- | --- | --- | --- |
| Gene ID | Gene name | S1_FPKM | S2_FPKM | S3_FPKM | S1_*vs*_S2 | S2_*vs*_S3 | Description |
|  |  |  |  |  | Log2 (Fold_change) | Log2 (Fold_change) |  |
| LOC105041929 | *SAPK2* | 14.01 | 2.10 | 1.12 | -2.74 | – | serine/threonine-protein kinase |
| LOC105048639 | *SAPK1* | 33.80 | 2.05 | 1.86 | -4.04 | – | serine/threonine-protein kinase |
| LOC105058236 | *ABF2* | 56.95 | 13.86 | 21.40 | -2.04 | – | bZIP transcription factor |
| LOC105060907 | *ABF2* | 12.46 | 3.70 | 5.36 | -1.75 | – | bZIP transcription factor |
| LOC105047693 | *PP2C06* | 4.43 | 1.41 | 1.71 | -1.66 | – | protein phosphatase |
| LOC105051995 | *BZIP12* | 6.45 | 2.59 | 3.02 | -1.31 | – | bZIP transcription factor |
| MSTRG.598 | *PP2C06* | 19.95 | 3.85 | 5.96 | -2.37 | – | protein phosphatase |
| LOC105044347 | *PP2CA* | 146.12 | 70.32 | 81.94 | -1.06 | – | protein phosphatase |
| LOC105052394 | *DPBF3* | 2.99 | 0.96 | 1.26 | -1.64 | – | abscisic acid-insensitive 5-like protein |
| LOC105045895 | *PYL4* | 0.15 | 0.93 | 0.76 | 2.63 | – | abscisic acid receptor |
| LOC105053864 | *PYL1* | 0.08 | 0.43 | 0.34 | 2.43 | – | abscisic acid receptor |
| LOC105043050 | *AHG1* | 56.61 | 118.32 | 115.76 | 1.06 | – | protein phosphatase |
| LOC105052243 | *PYL4* | 0.12 | 0.69 | 0.72 | 2.52 | – | abscisic acid receptor |
| LOC105048010 | *PYL2* | 0.21 | 0.60 | 2.96 | 1.49 | 2.30 | abscisic acid receptor |
| LOC105037142 | *PYL10* | 0.27 | 1.13 | 2.77 | 2.06 | – | abscisic acid receptor |
| LOC105038096 | *DPBF3* | 1.32 | 3.72 | 6.14 | 1.49 | – | abscisic acid-insensitive 5-like protein |

| **Additional file 4: Table S1-h** Differentially expressed genes involved in ABA signal transduction during zygotic embryo development of oil palm. | | | | | | | |
| --- | --- | --- | --- | --- | --- | --- | --- |
| Gene ID | Gene name | S1_FPKM | S2_FPKM | S3_FPKM | S1_*vs*_S2 | S2_*vs*_S3 | Description |
|  |  |  |  |  | Log2 (Fold_change) | Log2 (Fold_change) |  |
| LOC105053512 | *CYP707A5* | 18.41 | 4.57 | 11.85 | -2.01 | 1.37 | abscisic acid 8'-hydroxylase |
| LOC105033850 | *CYP707A5* | 5.58 | 1.19 | 0.54 | -2.23 | -1.14 | abscisic acid 8'-hydroxylase |
| LOC105043314 | *CA2* | 24.48 | 5.88 | 6.26 | -2.06 | – | beta-carotene 3-hydroxylase |
| LOC105060532 | *LCY1* | 4.11 | 1.64 | 1.95 | -1.33 | – | lycopene beta cyclase |
| LOC105035937 | *AO1* | 13.05 | 2.36 | 3.82 | -2.47 | – | indole-3-acetaldehyde oxidase-like |
| LOC105036563 | *NCED1* | 0.14 | 0.39 | 0.26 | 1.43 | – | 9-cis-epoxycarotenoid dioxygenase |
| LOC105045553 | *ZSD1* | 0.17 | 2.50 | 5.56 | 3.85 | – | zerumbone synthase |
