# Supplementary material for: Transcriptome analysis reveals key developmental and metabolic regulatory aspects of oil palm (Elaeis guineensis Jacq.) during zygotic embryo development
Source: BMC Plant Biol. 2022 Mar 12;22:112. doi: 10.1186/s12870-022-03459-2 (PMC8917659; doi:10.1186/s12870-022-03459-2)
Supplement: Supplementary file 5 — Additional file 5: Table S2. Differentially expressed genes involved in starch and sucrose metabolism during zygotic embryo development of oil palm. [file 12870_2022_3459_MOESM5_ESM.docx]

| **Additional file 5: Table S2** Differentially expressed genes involved in starch and sucrose metabolism during zygotic embryo development of oil palm. | | | | | | | |
| --- | --- | --- | --- | --- | --- | --- | --- |
| Gene ID | Gene name | S1_FPKM | S2_FPKM | S3_FPKM | S1_*vs*_S2 | S2_*vs*_S3 | Description |
|  |  |  |  |  | Log2 (Fold_change) | Log2 (Fold_change) |  |
| LOC105045149 | *SUS4* | 47.52 | 10.60 | 5.92 | -2.16 | – | sucrose synthase |
| LOC105053047 | *SUS3* | 6.27 | 1.53 | 0.70 | -2.03 | -1.13 | sucrose synthase |
| LOC105057453 | *SUS4* | 27.17 | 78.39 | 79.86 | 1.53 | – | sucrose synthase |
| LOC105058592 | *SUS1* | 20.20 | 160.20 | 213.98 | 2.99 | – | sucrose synthase |
| LOC105059813 | *malZ* | 0.42 | 0.92 | 1.75 | 1.13 | – | alpha-glucosidase |
| LOC105052110 | *CIN3* | 0.02 | 0.16 | 0.91 | 3.00 | 2.51 | beta-fructofuranosidase |
| LOC105050124 | *SPS1* | 38.73 | 9.07 | 10.17 | -2.09 | – | sucrose-phosphate synthase |
| LOC105039053 | *SPS1* | 35.62 | 9.17 | 11.14 | -1.96 | – | sucrose-phosphate synthase |
| LOC105049657 | *SPS2* | 8.88 | 3.23 | 5.84 | -1.46 | – | sucrose-phosphate synthase |
| LOC105037731 | *WAXY* | 2.75 | 0.84 | 1.06 | -1.71 | – | granule-bound starch synthase |
| LOC105059288 | *WAXY* | 0.87 | 30.49 | 72.09 | 5.13 | 1.24 | granule-bound starch synthase |
| LOC105035803 | *APS2* | 0.49 | 0.05 | 0.08 | -3.39 | – | inactive glucose-1-phosphate adenylyltransferase small subunit |
| LOC105047182 | *AGPS1* | 72.74 | 29.83 | 56.39 | -1.29 | – | glucose-1-phosphate adenylyltransferase large subunit |
| LOC105048507 | *AGPS1* | 0.67 | 4.76 | 7.88 | 2.82 | – | glucose-1-phosphate adenylyltransferase large subunit |
| LOC105050621 | *AGPP* | 0.04 | 0.22 | 0.77 | 2.32 | – | glucose-1-phosphate adenylyltransferase small subunit |
| LOC105035295 | *SS3* | 5.19 | 2.09 | 3.91 | -1.31 | – | starch synthase |
| LOC105048676 | *SS4* | 2.90 | 0.43 | 0.32 | -2.74 | – | starch synthase |
| LOC105037731 | *WAXY* | 2.75 | 0.84 | 1.06 | -1.71 | – | granule-bound starch synthase |
| LOC105059288 | *WAXY* | 0.87 | 30.49 | 72.09 | 5.13 | 1.24 | granule-bound starch synthase |
| LOC105042584 | *AMY2* | 6.75 | 2.91 | 4.48 | -1.21 | – | alpha-amylase |
| LOC105058203 | *AMY3* | 0.02 | 0.15 | 0.63 | 2.87 | – | alpha-amylase |
| LOC105043558 | *glpV* | 0.80 | 5.30 | 9.64 | 2.73 |  | glycogen phosphorylase |
| LOC105057692 | *DPE2* | 2.24 | 4.57 | 7.52 | 1.03 |  | 4-alpha-glucanotransferase |
| LOC105041676 | *DPE1* | 34.70 | 104.65 | 117.88 | 1.59 |  | 4-alpha-glucanotransferase |
