# Supplementary material for: Transcriptome analysis reveals key developmental and metabolic regulatory aspects of oil palm (Elaeis guineensis Jacq.) during zygotic embryo development
Source: BMC Plant Biol. 2022 Mar 12;22:112. doi: 10.1186/s12870-022-03459-2 (PMC8917659; doi:10.1186/s12870-022-03459-2)
Supplement: Supplementary file 6 — Additional file 6: Table S3. Differentially expressed genes involved in fatty acid biosynthesis during zygotic embryo development of oil palm. [file 12870_2022_3459_MOESM6_ESM.docx]

| **Additional file 6: Table S3** Differentially expressed genes involved in fatty acid biosynthesis during zygotic embryo development of oil palm. | | | | | | | |
| --- | --- | --- | --- | --- | --- | --- | --- |
| Gene ID | Gene name | S1_FPKM | S2_FPKM | S3_FPKM | S1_*vs*_S2 | S2_*vs*_S3 | Description |
|  |  |  |  |  | Log2 (Fold_change) | Log2 (Fold_change) |  |
| LOC105053059 | *KAS12* | 24.83 | 9.77 | 13.37 | -1.34 | – | 3-oxoacyl-[acyl-carrier-protein] synthase |
| LOC105056688 | *KAS2* | 0.32 | 1.98 | 4.38 | 2.61 | – | 3-oxoacyl-[acyl-carrier-protein] synthase |
| LOC105040922 | *KAS1* | 0.40 | 5.39 | 7.60 | 3.76 | – | 4-oxoacyl-[acyl-carrier-protein] synthase |
| LOC105042279 | *FabG* | 0.34 | 0.94 | 1.37 | 1.49 | – | NADPH-dependent aldehyde reductase-like protein |
| LOC105042280 | *FabG* | 0.26 | 5.77 | 25.39 | 4.45 | 2.14 | NADPH-dependent aldehyde reductase-like protein |
| LOC105038379 | *fabZ* | 0.58 | 7.23 | 11.20 | 3.64 | – | 3-hydroxyacyl-[acyl-carrier-protein] dehydratase |
| LOC105032448 | *FAB2* | 184.59 | 55.15 | 32.70 | -1.74 | – | stearoyl-[acyl-carrier-protein] 9-desaturase |
| LOC105053527 | *SAD3* | 4.08 | 24.96 | 64.11 | 2.61 | 1.36 | stearoyl-[acyl-carrier-protein] 9-desaturase |
| LOC105053553 | *S-ACP-DES5* | 0.12 | 2.01 | 5.02 | 4.06 | – | stearoyl-[acyl-carrier-protein] 9-desaturase |
| LOC109505499 | *FATA* | 0.03 | 0.55 | 0.72 | 4.21 | – | oleoyl-acyl carrier protein thioesterase |
