# Supplementary material for: Transcriptome analysis reveals key developmental and metabolic regulatory aspects of oil palm (Elaeis guineensis Jacq.) during zygotic embryo development
Source: BMC Plant Biol. 2022 Mar 12;22:112. doi: 10.1186/s12870-022-03459-2 (PMC8917659; doi:10.1186/s12870-022-03459-2)
Supplement: Supplementary file 7 — Additional file 7: Table S4. Comparing differential expression genes from RNA-seq and qRT-PCR during zygotic embryo development of oil palm. [file 12870_2022_3459_MOESM7_ESM.docx]

| **Additional file 7: Table S4** Comparing differential expression genes from RNA-seq and qRT-PCR during zygotic embryo development of oil palm. | | | | | |
| --- | --- | --- | --- | --- | --- |
| Gene ID | Gene name | Development stage | RNAseq | qRT-PCR | Decision coefficient (R2) |
| LOC105040857 | *IAA17* | S1 | 49.6667 | 1.0052 | 0.9244 |
|  |  | S2 | 155.7367 | 1.8749 |  |
|  |  | S3 | 303.1700 | 6.3835 |  |
| LOC105032028 | *PIL13* | S1 | 142.8600 | 1.0004 | 0.9195 |
|  |  | S2 | 49.7267 | 0.5131 |  |
|  |  | S3 | 39.3800 | 0.2226 |  |
| LOC105041269 | *ALC2* | S1 | 916.4167 | 1.0079 | 0.9937 |
|  |  | S2 | 149.3700 | 0.0637 |  |
|  |  | S3 | 77.7800 | 0.0666 |  |
| LOC105045149 | *SUS4* | S1 | 47.5167 | 1.0040 | 0.9467 |
|  |  | S2 | 10.6000 | 0.0210 |  |
|  |  | S3 | 5.9233 | 0.1591 |  |
| LOC105053527 | *SAD3* | S1 | 4.0767 | 1.0222 | 0.9025 |
|  |  | S2 | 24.9567 | 2.8520 |  |
|  |  | S3 | 64.1067 | 51.2076 |  |
| LOC105058236 | *ABF2* | S1 | 56.9500 | 1.0029 | 0.9996 |
|  |  | S2 | 13.8600 | 0.1674 |  |
|  |  | S3 | 21.4033 | 0.3292 |  |
